# Emergency Medicine in the Kingdom of Bahrain

**DOI:** 10.1186/s12245-018-0163-1

**Published:** 2018-02-08

**Authors:** Feras Abuzeyad, Leena Alqasem, Mudhaffar I. Al Farras, Shaikha S. Al Jawder, Ghada Al Qasim, Salah Alghanem

**Affiliations:** 1 Department of Emergency Medicine, King Hamad University Hospital, Building 2345, Road 2835, Block 228, P. O. Box 24343, Busaiteen, Kingdom of Bahrain; 2 National Health Regulatory Authority, Seef, Kingdom of Bahrain; 3 Emergency Medicine Department–Royal Medical Services, Bahrain Defence Force, Riffa, Kingdom of Bahrain

**Keywords:** Kingdom of Bahrain, Emergency medicine, Emergency medical services

## Abstract

It has been more than a decade since emergency medicine became recognized as a specialty in the Kingdom of Bahrain. In the last fifteen years emergency medicine has widely established itself and developed rapidly in the Kingdom. The three main emergency departments are: Salmanyia Medical Complex (SMC), Royal Medical Services of Bahrain Defence Force (RMS-BDF) and King Hamad University Hospital (KHUH) are now fully equipped and operated by a majority of board certified emergency physicians.

Standardized protocols, and the Central National Ambulance will be established in the near future, and the ambulances will offer both basic and advanced life support by trained nurses and paramedics.

Emergency Medicine residency training programs were established in the main three hospitals in Bahrain for the Arab Board Certification initially, while currently only two hospitals, BDF hospital and KHUH are recognized as training centers for the Saudi Board Residency Program.

This article will focus on many aspects related to emergency medicine in the Kingdom of Bahrain including: history of health care systems in Bahrain, hospitals and primary care, disaster management, Emergency medical services (EMS), hospital-based emergency care, training in emergency medicine and universities. We aim to present Bahrain’s past and existing emergency medicine experience, our perspective about the existing challenges faced by the specialty, and the future plans for the advancement of emergency medicine in the Kingdom.

## Background/demographics

**Fig. 1:**
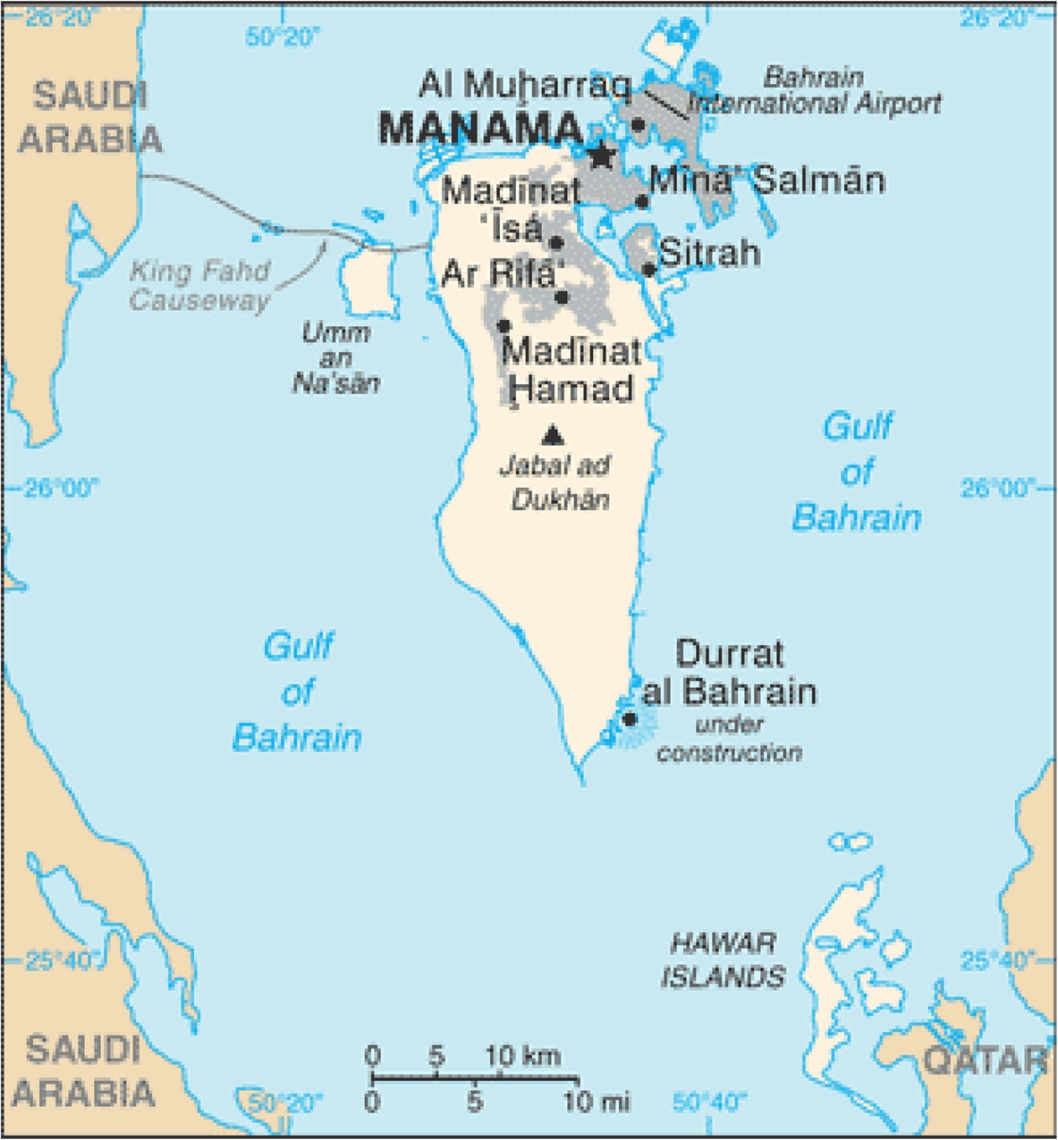
Kingdom of Bahrain among Arab Gulf countries

**Fig. 2:**
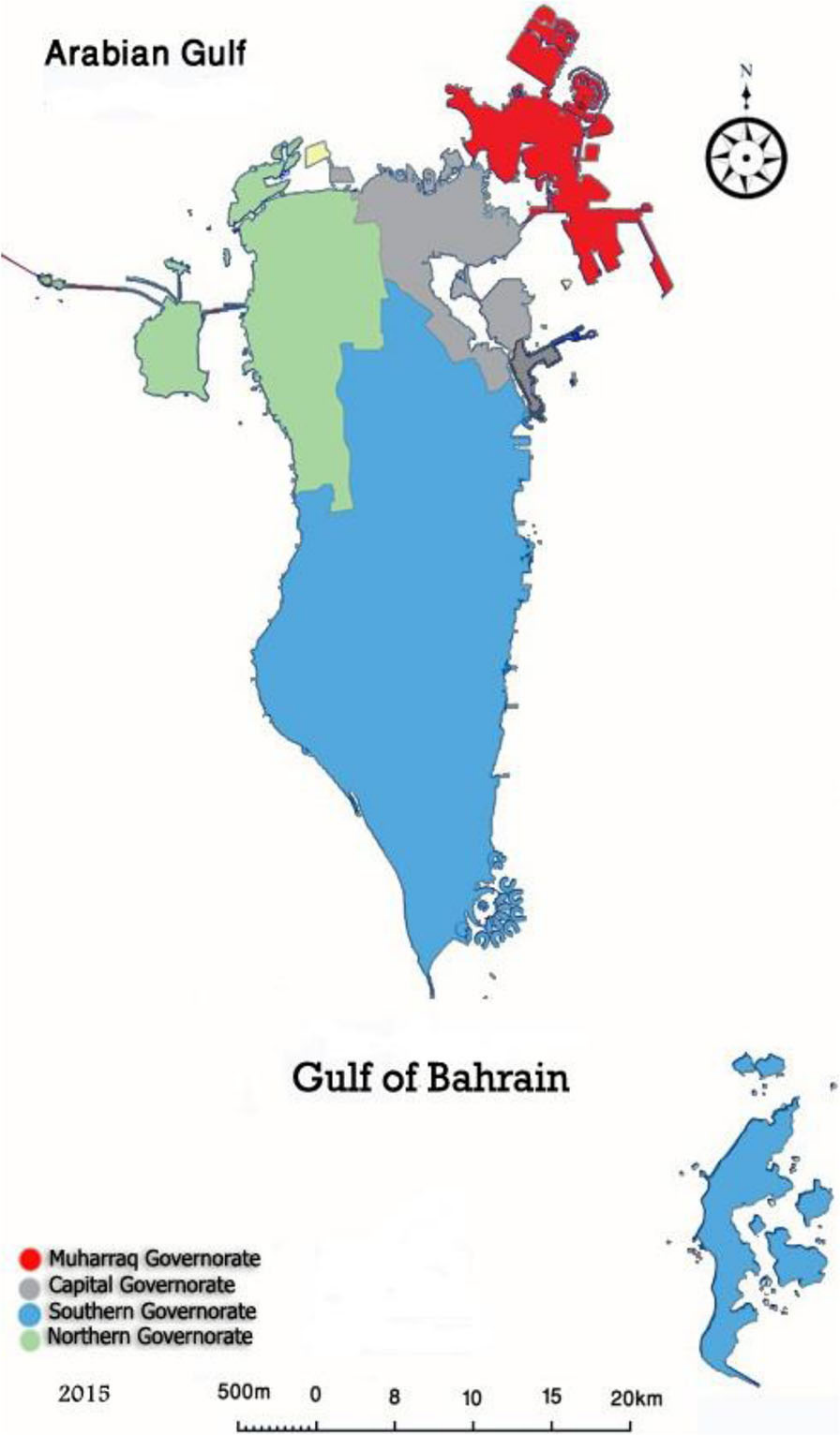
The four governorates of the Kingdom of Bahrain

The Kingdom of Bahrain is an archipelago made up of 33 islands. It is located in the Arabian Gulf on the east coast of the Kingdom of Saudi Arabia (Fig. 1). Bahrain has a total area of 770.9 km^2^. The Kingdom comprises of four governorates: Al-Muharraq, Capital, Southern, and Northern (Fig. 2), and according to the Central Informatics Organization has a population of 1,315,000 with a population density of 1705 person/km^2^ [1, 2].

In 2015, Bahrain was ranked 45 as a country with very high human development (the highest of four categories) on the United Nations Development Program’s (UNDP’s) Human Development Index (HDI) [3]. Net migration to Bahrain in 2012 was 14.74 migrants per 1000 populations, the eighth highest in the world; the 2012 population growth rate of 2.64% was the 20th highest in the world, the total fertility rate was 1.9 children per women, and the birth rate was 14.4 births per 1000 populations [4].

According to the World Bank report, the Kingdom’s health care costs accounted for 4.9% of Gross Domestic Product (GDP) in 2013. The report also noted the improvement in life expectancy at birth, which improved from 71 in 1983 to 77 in 2013. Similarly, infant mortality dropped from 23 per 1000 births in 1983 to 5 per 1000 births in 2013 [5].

## History of health care in Bahrain

Bahrain became a British protectorate in the nineteenth century and become independent in 1971. Bahrain has one of the oldest health care systems in the Gulf, and the country hosts the oldest hospital in the region—the American Mission Hospital (AMH), which was founded in 1902 by the Reform Church of America. The hospital provided care for Bahrainis as well as travelers from neighboring countries. In 1925, the government established a small clinic in a shop with a single Indian doctor to mainly treat injured pearl divers. Within the same year, the Public Health Directorate was established. A small police force hospital was established in 1936 and was converted to an isolation ward the next year, which remained open until 1941 [6, 7].

Similar to other Gulf Cooperation Council (GCC) countries, Bahrain developed a comprehensive health care system financed by oil revenues [7]. This was initiated by the establishment of Awali Hospital, a hospital with a 37-bed capacity, which was governed and owned by the Bahrain Petroleum Company. The first formal governmental hospital, Al Noaim Hospital, was inaugurated between 1940 and 1942. Due to the increase in the size of the population, the Salmaniya Medical Complex was also established in 1957. Subsequently, the population of Bahrain continued to increase due to the prevention of the spread of infectious diseases and the improvement of the general health of the population. Furthermore, Bahrain joined the World Health Organization (WHO) in 1967 [6].

## Health care system

Bahrain has a network of numerous medical facilities in both the public and private sectors. It provides comprehensive health care services to the whole population, including expatriates (non-nationals) in compliance with the WHO global objectives. Nationals receive all health care services for free by the Ministry of Health, while expatriates pay nominal fees for the services received [6].

The Primary Health Care services represent the cornerstone of health care provision and are provided through 27 health centers functioning under the governance of the Ministry of Health [2], which offer a wide range of curative, preventive, and supportive services [8] by family physicians and general practitioners. Secondary and tertiary health care services are provided through three main governmental hospitals: Salmaniya Medical Complex, Bahrain Defense Force Hospital, and King Hamad University Hospital. These hospitals are fully equipped, with corresponding ambulance services and helipads.

The Ministry of Health also provides specialized services through other hospitals, including one maternity hospital, one psychiatric hospital, and one geriatric hospital. In addition, there are also more than 16 private hospitals across the island, where patients pay for the health care services received either individually or via their medical insurance.

## Health care regulation

The task of regulating the health care sector was transferred from the Ministry of Health to the National Health Regulatory Authority (NHRA) in 2009. The NHRA is responsible for regulating the health care sector in the Kingdom of Bahrain through the licensing and accrediting of health care facilities, licensing health care professionals, registration and pricing of pharmaceuticals, licensing drug manufacturers, granting approvals for clinical trials, investigating complaints, and conducting disciplinary hearings for health care professionals [9].

The NHRA recognizes emergency medicine as a primary specialty and physicians are only licensed as specialists in that field if they hold recognized qualifications in emergency medicine from American, Canadian, Saudi, or Arab boards, or other equivalent Boards in emergency medicine.

According to the Supreme Council for Health Decision No. (40) for the year 2016, regarding the duration of validity of licenses and the conditions for their renewal, all specialized physicians are required to accumulate a minimum of 30 Continuous Professional Development (CPD) hours annually prior to their licenses being renewed [10].

To further emphasize the importance of an independent health regulator, the Supreme Council of Health (SCH) became the Board of directors of NHRA in 2015. The SCH was established in 2012, and it is responsible, among many other items, for formulating national policies regarding the total number of hospital beds required in the Kingdom of Bahrain; embarking and mandating a policy for financing health care services through the enforcement of a Health Insurance System; endorsing standardized policies for health professionals training in hospitals; and estimating the distribution of health care facilities throughout the kingdom [5].

## Salmaniya Medical Complex (SMC)

The Salmaniya Medical Complex is the largest health care facility in the kingdom [11], which was established in 1957 with 50-bed capacity, but was substantially expanded in 1978 to reach a bed capacity of 926 [12]. The hospital provides emergency, secondary, and tertiary health care services and hosts a number of specialized outpatient clinics. The Hereditary Blood Disorders Centre opened at the SMC in 2014, providing 90 beds for patients suffering from a variety of hematological conditions, including sickle cell disease among others. SMC functions as a teaching and research center and is affiliated with the Arabian Gulf University.

The Department of Accident and Emergency at SMC is the largest with a bed capacity of 85, with annual census of approximately 300,000 patients per year. The department uses the Manchester Triage System. The department is prepared with up-to-date equipments and is a recognized training center of the Arab Board of Emergency Medicine (ABEM).

## Bahrain Defence Force Hospital—Royal Medical Services

BDFH is the second largest hospital in the country. The hospital was established in 1979 with a bed capacity of 120, providing health care services to the Ministry of Defense and Ministry of Interior personnel and their dependents [13]. In 1992, the hospital was expanded to have a bed capacity of 400, with the inauguration of Mohammed bin Khalifa bin Salman Cardiac Centre as a national project to provide comprehensive adult and pediatric diagnostic and therapeutic cardiac care. Within that spectrum, the center provides interventional and surgical cardiac services including open and closed cardiac surgery procedures [14].

The hospital is affiliated with the Arabian Gulf University and the Royal College of Surgeons in Ireland (RCSI)–Medical University of Bahrain as a teaching institution.

In 1979, the Emergency Department (ED) was operating as Casualty and Out Patient Clinic. In 2010, the department’s name was changed to the Emergency Medicine Department (EMD). The EMD provide 24-h healthcare services by qualified emergency physicians to individuals presenting with various types of emergencies, with an annual census of 100,000 patients per year.

The department has 31 beds, for both adult and pediatric patients, and a short stay unit of 11 beds capacity. The department utilizes the Canadian Triage Acuity Scale. The department is recognized as a training center for emergency medicine for both Arab Board of Emergency Medicine (ABEM) and the Saudi Board of Emergency Medicine (SBEM) programs.

## King Hamad University Hospital

King Hamad University Hospital (KHUH) was established by a Royal Decree in 2010, as one of the state of the art hospitals with modern technology and elegant design. It has a capacity of 311 beds; 110 beds will be added once the National Oncology & Research Center is inaugurated and fully functional by the end of 2017 [15].

The hospital possesses one of the largest Hyperbaric Oxygen units in the Middle East, which is equipped with eight single champers and one large multi-chamber. The hospital also introduced its new Simulation Center on April 2016 [16]. KHUH is a university hospital affiliated with Royal College of Surgeons in Ireland (RCSI)–Medical University of Bahrain.

The Emergency Medicine Department in KHUH has a total of 45 beds with state-of-the-art equipments and sees around 90,000 patients a year, both adult and pediatric. The triage system utilized is the Manchester Triage System. The department is recognized as a training center for emergency medicine for both Arab Board of Emergency Medicine (ABEM) and the Saudi Board of Emergency Medicine (SBEM) programs.

## Private hospitals

There are 19 private hospitals spread throughout the Kingdom; however, most are concentrated in and around the capital, Manama. These hospitals provide a variety of services with capacities of less than 50 beds.

The private hospitals either have an emergency room or a 24-h clinic operated by general practitioners or physicians with clinical experiences in emergency medicine. These units act more as urgent care units rather than emergency departments where most cases are stabilized and later transported to one of the three governmental hospitals [17].

## Insurance system

Medical service is free including immunization, outpatient treatment, and hospitalization, and there is provision for most forms of social security, including pensions, sick pay, compensation for work injury, unemployment benefits, and maternity and family allowance payments. As the cost of health care services is increasing steadily, and in alliance other gulf states, Bahrain is planning to shift from a state-sponsored health care system to a national health insurance system. The new insurance system aims to provide the freedom for the recipients of health care services to select the health care provider from both the public and private hospitals.

## Pre-hospital emergency care

In the year of 1979, a senior orthopedic surgeon, Lt. Col. Dr. Ali Al-Kahlifa, published an article which proposed the need to establish an Emergency Medical Service (EMS) in Bahrain [18], and it was not until 1985 when the EMS was established within the SMC.

According to the World Health Organization (WHO), as of 2007, Bahrain had a formal and publicly available emergency care system (pre-hospital care), accessible through a universal national access number [4]. Road traffic accidents’ fatality rates per 100,000 population in Bahrain are 12.3 [19].

In Bahrain, the EMS coverage is hospital based and shared by the ambulance departments of the three major hospitals; Salmaniya Medical Center, Bahrain Defense Force Hospital, and King Hamad University Hospital as part of a nationwide integrated public service.

The EMS system in the Kingdom of Bahrain follows the Anglo-American model. The ambulances are staffed with a nurse/paramedic who is trained in Basic Life Support, Advanced Cardiac Life Support, Pre-Hospital Trauma Life Support, and Pediatric Advanced Life Support, and a driver with ambulance driving license and first aid training. They provide out of hospital stabilization and transportation of patients to the hospital for further management (load and go concept).

This service is free of charge and can be accessed by dialing the national ‘999’ emergency phone number. A centralized call center communicates with ambulance services in the designated hospital to dispatch the ambulances.

The EMS in the three hospitals involves active planning as stand-by in major events coverage, which often requires special logistics and organization so that normal operation is not adversely affected. Examples of such events include Formula One car race, Iron Man, Triathlon race, and other major sporting event where there is a significant mass gathering.

Taking into account the rapid population growth, there was a need to expand the EMS capacity, and thus, the Central National Ambulance project was proposed in 2011.This is a nationwide integrated EMS plan proposed to cover the entire kingdom’s population. It will include 13 satellite ambulance dispatch stations across Bahrain, which is linked with the main operating room of the National Ambulance Centre at the Interior Ministry, which is equipped with high-tech devices and communication systems to communicate and monitor the ambulance fleet through the GPS system [20]. The expanded service is expected to speed up emergency response times nationwide in more effective and efficient manner, and this will lead to a higher quality of care and lower mortality in emergency cases.

## Disaster planning

The occurrence of both natural and man-made disasters shifted attention to disaster and emergency medicine, granting them a priority in health care services [21].

In 2009, Dr. Abdul Aziz Hamza published his book “Tears on an Island: A History of Disasters in the kingdom of Bahrain” which covers various disasters in and around Bahrain from the late 1800s up until early twenty-first century.

The first reported disaster in Bahrain was the cholera epidemic in 1904. This cholera outbreak killed more than 2000 people at a time when the population of Bahrain was only 30,000. The disease also infected 5000 people. That year became known as the Year of Mercy. Other more recent reported disasters include the Gulf Air plane crash which occurred on August 23, 2000, killing 143 people and Al Dana dhow tragedy on March 30, 2006, killing 58 people [22].

After all these tragic crises, the authorities in the kingdom established the National Commission for Disaster Management (NCDM) in May 2006. NCDM is headed by Chief of Public Security, with members from the following institutions: Civil Defense, Customs Affairs, Municipality, Health Ministry, Electricity & Water, Works & Housing, Information, Finance, Bahrain Defense Force, National Guard, and Civil Aviation Authority. Its main duty was the responsibility of coordination between the different member organizations. In addition, the NCDM is responsible for the assurance of the vigilance and alertness of these organizations [23].

The Gulf Cooperation Council (GCC) countries agreed to establish the GCC Emergency Management Center (GCC EMC) in 2013. It aims to enhance regional incident response and promote integration for increased coordination, convergence, and integration among member countries. It also aims to increase the number of qualified personnel in emergency medicine through the promotion of candidates to become academically qualified in emergency management. As a step to achieve this goal, the GGC EMC reached an agreement with Georgetown University and established a new executive master’s degree program tailored to meet the needs of GCC emergency and disaster management [24].

## EM specialty

Since their establishment, all the tertiary hospitals in Bahrain have an Emergency Department (ED) or Accident and Emergency Department. This is in line with the growing recognition of the importance of emergency medicine and its efficacy in the provision of health care services [25]. NHRA mandates the existence of an emergency department in all hospitals. The exact date of specialty recognition is unknown; however, in the early 2000s, the graduates in the specialty who had a recognized certified board in emergency medicine had no obstacle to be recognized and be appointed as full-time consultants in the ED (Table 1). Earlier to this date the ED’s were staffed with general medical officers with backup and support from others on call specialties in the hospital, and the director of the department used to be a general or an orthopedic surgeon. Compared to other countries, the specialty faced similar challenges and obstacles in its development, but now it is recognized by NHRA as an independent and respected specialty.

**Table 1:** Graduates in the specialty who had a recognized certified board in emergency medicine

Year of graduation	Board certification	Certifying body
2003: 1st Bahraini physician	Fellow of Royal College of Physicians	Royal College of Physicians and Surgeons of Canada
2005: 1st Bahraini physician	Saudi Board of Emergency Medicine (SBEM)	Saudi Commission for Health Specialties
2005: 1st three Bahraini physicians	Arab Board of Emergency Medicine (ABEM)	Arab Board of health Specializations Council
2009: 1st Bahraini physician	Saudi Board of Pediatric Emergency Medicine (SBEM)	Saudi Commission for Health Specialties

According to the 2016 National Health Regulatory Authority annual report, there is a total of 19 registered emergency medicine physicians, 8 of them are board certified. However, there is no projection as to the number of Emergency Physicians required to address the burden of emergencies in the Kingdom [26].

## Training programs in EM

Bahrain depends heavily on two main training programs in EM, the Arab and Saudi Boards. In 1979, the 17 Arab country members of the Supreme Council approved the By-laws of the Arab Board of Medical Specialties [27]. The Arab Board of Emergency Medicine (ABEM) program is accredited by the Arab Board of health Specializations Council.^1^ It is a 5-year program whereby the physicians enrolled into the program in one of the three recognized tertiary centers.

The Saudi Board of Emergency Medicine (SBEM) program which is accredited by the Saudi Commission for Health Specialties is a 4-year residency program [28], and only the BDFH and KHUH are recognized training centers in this program.

Currently, many of the early graduates of the two programs hold critical and important positions in EDs in the kingdom.

Arab Board training in Emergency Medicine program was initiated by Ministry of Health through the Arab League for Medical Specializations and their recognition of its secondary care hospital as a training center for that program. Later on, other hospitals namely, the Bahrain Defense Force Hospital and King Hamad University Hospital, gained recognition as training centers for the Saudi Board training in Emergency Medicine through the Saudi Commission for Health Specialties. There is an initiative currently to combine all governmental hospitals as one training center for all specialization programs including Emergency Medicine.

## Medical universities

There are two medical universities in Bahrain namely, the Arabian Gulf University (AGU) and the Royal College of Surgeons in Ireland–Medical University of Bahrain (RCSI-MUB).

The Arabian Gulf University was founded in 1979, as a Gulf Council Cooperation (GCC) institution based on the belief of the importance of the cooperation between gulf countries to fulfill the need of medical experts in the region. The university uses problem-based learning with six-year program. It admits up to 150 students annually from all GCC countries [29].

As part of Bahrain’s strategic vision to establish a center for international education and training, the Bahraini Government invited the Royal College of Surgeons in Ireland to establish a center for medical and para-medical education and training in Bahrain. The Royal College of Surgeons in Ireland-Medical University in Bahrain (RCSI-MUB) was founded in 2004. The college has 6-year program and admits international students in addition to GCC residents. The first group of students graduated from the college in June 2010, with degrees recognized by the National University of Ireland [30].

The emergency medicine rotation has been introduced in the 2 existing medical universities: RCSI–MUB and AGU. Though it is a short 1-week rotation with emergency-focused didactic lectures, but it gives an exposure to emergency practice. Students are introduced to common clinical scenarios encountered in the ED practice. Many of the emergency consultants are clinical lecturers in the two mentioned universities.

## Nursing schools/nursing training in emergency

Two universities provide undergraduate nursing degrees the kingdom for nursing, the University of Bahrain and the Royal College of Surgeons in Ireland-Medical University of Bahrain. The College of Health Sciences (CHS), in collaboration with the American University of Beirut and The University of Illinois at Chicago, was established in 1976 under the administrative authority of the Ministry of Health. In 2011, the administrative authority was shifted from the Ministry of Health to the University of Bahrain. The CHS is conveniently located on the campus of Salmaniya Medical Complex, giving its students easy access to the hospital allowing them to spend considerable time in the various departments of the hospital [31]. The College of Health Sciences also provides a 1-year specialization program in emergency nursing.

The Royal college of Surgeons established its Bachelor nursing program in 2009 [32]. Student nurses from RCSI are allocated in governmental hospitals such as the Bahrain Defense Force Hospital and King Hamad University Hospital in order to enhance their clinical development. The Master Nursing program has been developed with clinical nursing practice at its core [33].

## Bahrain Association of Emergency Medicine

The Bahrain Emergentologist Association (BEMASSO) was established in 2004 under the governance of the Bahrain Medical Society, which was founded in 1970. In 2014, BEMASSO had its title changed to Bahrain Association of Emergency Medicine (BAEM) in official elections run by the board of directors. BAEM was the first Arab country to join the International Federation of Emergency Medicine (IFEM) as a full member in 2006 [34].

BAEM has also joined the Gulf Federation of Emergency Medicine (GFEM), and the Kingdom of Bahrain has been chosen to be the first of GCC to lead GFEM and host the first General Secretary. The association plays a major role in promoting and advancing the specialty in the kingdom and the region and succeeded in gaining fame and respect within the medical community.

BAEM functions under the broader umbrella of the Bahrain Medical Society (BMS), which is a social professional entity governed by the Ministry of Social Development. Currently, BAEM has 31 registered members. BAEM has no licensing or certification role in emergency medicine in the Kingdom as this is the sole role of the National Health Regulatory Authority (NHRA).

## Medical journals in Bahrain

There is no specialty medical journal; however, there are two medical journals in Bahrain which publish articles focusing on emergency medicine. The first journal is the Bahrain Medical Bulletin. This journal is published on a quarterly basis of each year. It is one of the oldest medical journals in the Middle East, and the first issue was released in July 1979 [35]. The journal is indexed in the Medicus for Eastern Mediterranean Region (IMEMRI) of the World Health Organization Index. The journal is also Extramed of the United Kingdom and International Serial Data System of France.

The second journal is the Journal of the Bahrain Medical Society, which was established in 1994 with quarterly publications.

## Challenges/future

The challenges confronting emergency medicine in the Kingdom of Bahrain were almost similar to those of the other GCC countries currently and in North America 35 years ago when they started establishing emergency medicine as a specialty. One of the major challenges is the severe shortage of well-trained, board-certified emergency physicians in the kingdom that affects the quality of care provided. The unavailability of board-certified consultants in all shifts does affect patients’ management and disposition. The shortage of residents interested in emergency medicine reflects largely on the training program and the total number of graduates each year. One of the reasons for this lack of interest is compensation issues such as salary & lack of competitive pay of the qualified Emergency physicians who continue to shift base duties in an acute critical area in comparison to other less stressful specialties. Emergency medical services play a vital role in the success of patient care before the arrival to an emergency department, which will affect the patient’s outcome. The National Emergency Medical Services project is being developed to improve and standardized the pre-hospital care to the international levels. Authorities and the public underestimate the importance and value of emergency medicine which consequently accumulates pressure on the qualified physician to change these views and pass a strong message that emergency medicine is a specialty on its own and is needed in all fields and events.

## Future plans

In the Kingdom of Bahrain, emergency medicine specialty when compared to others is still a young specialty; however, it succeeded to establish itself and set the standards of care in both the emergency field and prehospital care services.

We passed the struggling era which the specialty faced in some other parts of the world, and we proved ourselves as an effective specialty that others trust and continue to work with to improve the healthcare system. The training in emergency medicine is well established through the Saudi and Arab Board residency programs, and the number of graduates is increasing year after year.

Some areas are still needed to be enhanced and developed, which includes mainly increasing the commitment of emergency physicians towards their specialty and investing in subspecialties training, encouraging and advancing research, and finally improving training by utilizing simulation centers.

## Conclusion

Although emergency medicine has been neglected for so many years, it is being gradually recognized as a specialty by the health care system in the Kingdom of Bahrain. Although it is still at an infant state, the introduction of residency training programs and board-certified emergency physicians has a tremendous impact on the emergency services in the kingdom. With time, we strongly feel that the importance of emergency medicine will gain more momentum and recognition and that the specialty will be ranked at the same level as other specialties, if not higher.

## Abbreviations

ABEM: Arab Board of Emergency Medicine
AGU: Arabian Gulf University
BAEM: Bahrain Association of Emergency Medicine
BDFH: Bahrain Defense Force Hospital
BEMASSO: The Bahrain Emergitologist Association
CHS: College of Health Sciences
ED: Emergency Department
EMC: Emergency Management Center
EMD: Emergency Medicine Department
EMS: Emergency medical services
GCC: Gulf Cooperation Council
GFEM: Gulf Federation of Emergency Medicine
KHUH: King Hamad University Hospital
MUB: Medical University in Bahrain
NCDM: National Commission for Disaster Management
NHRA: National Health Regulatory Authority
RCSI: Royal College of Surgeons in Ireland
RMS-BDF: Royal Medical Services of Bahrain Defence Force,
SBEM: Saudi Board of Emergency Medicine
SCH: Supreme Council of Health
SMC: Salmaniya Medical Complex
WHO: World Health Organization

## Footnotes

1 The foundations of the Arab Council for terms of reference of health in 1978 by a decision of the Council of Arab Ministers of Health of the Arab League and aims to improve health service in the Arab world by raising the level of scientific and practical in various disciplines do not make a profit.
