# Correction to: Emergency Medicine in the Kingdom of Bahrain

**DOI:** 10.1186/s12245-018-0177-8

**Published:** 2018-03-05

**Authors:** Feras Abuzeyad, Leena Alqasem, Mudhaffar I. Al Farras, Shaikha S. Al Jawder, Ghada Al Qasim, Salah Alghanem

**Affiliations:** 1 Department of Emergency Medicine, King Hamad University Hospital, Building 2345, Road 2835, Block 228, P. O. Box 24343, Busaiteen, Kingdom of Bahrain; 2 National Health Regulatory Authority, Seef, Kingdom of Bahrain; 3 Emergency Medicine Department–Royal Medical Services, Bahrain Defence Force, Riffa, Kingdom of Bahrain

## Correction

Following the publication of the original article [1], it was brought to our attention that author Leena Alqasem was erroneously included as Leena Alqusem.

The correct author’s surname is included in the author list of this ‘Correction’ and has been updated in the original article [1].
